# Emotion recognition dysfunction after anesthesia and cardiac surgery

**DOI:** 10.3389/fpsyg.2022.1001493

**Published:** 2022-11-16

**Authors:** Delin Zhang, Yi Shen, Zhiyun Chen, Yang Guo, Zaifeng Gao, Jian Huang, Xiqian Lu

**Affiliations:** ^1^Department of Anesthesiology, First Affiliated Hospital, School of Medicine, Zhejiang University, Hangzhou, China; ^2^Department of Psychology and Behavioral Sciences, Zhejiang University, Hangzhou, China; ^3^Department of Data and Information, The Children’s Hospital Zhejiang University School of Medicine, Hangzhou, China; ^4^Sino-Finland Joint AI Laboratory for Child Health of Zhejiang Province, Hangzhou, China; ^5^National Clinical Research Center for Child Health, Hangzhou, China; ^6^Institute of Psychology, Chinese Academy of Sciences, Beijing, China

**Keywords:** emotion recognition, biological motion, cardiac surgery, cognitive changes, anesthesia and surgery, social cognition

## Abstract

Cognitive dysfunction after anesthesia and surgery has long been recognized. Recently, researchers provided empirical evidence for social cognition dysfunction (SCD) after anesthesia and surgery. In the present study, we concentrated on the deficits in emotion recognition, one of the most important clinical perspectives in SCD, in patients who underwent cardiac surgery. Biological motion (BM) was considered as the stimulus of interest, and patients’ abilities of BM emotion perception and action perception before and after anesthesia and surgery were examined. In total, 60 adult patients (40–72 years old) completed the BM recognition task, which required them to label the types of actions and emotions of perceived BM. The results showed that while action perception remained intact after cardiac surgery, 18.3% of patients exhibited deficits in emotion perception, further confirming the existence of SCD after anesthesia and surgery.

## Introduction

Cognitive changes in patients after anesthesia and surgery have long been reported (Moller et al., 1998; Hovens et al., 2014; Evered et al., 2018), such as memory loss, inattention, and difficulties in information processing (Hovens et al., 2012; Glumac et al., 2019). These symptoms are summarized as delayed neurocognitive recovery (within 30 days after cardiac surgery) or postoperative cognitive dysfunction (1–2 months after cardiac surgery). However, almost all empirical evidence revealed the deficit of general cognitive function after anesthesia and surgery, and little is known about the influence of anesthesia and surgery on social cognition.

Humans are social species. Much of our behaviors are linked with social and emotional motivations. Social cognition concerns various psychological processes to perceive, manipulate, and interpret social information (Henry et al., 2016), which is vital to improving quality of life (Hasson-Ohayon et al., 2017; Yogarajah and Mula, 2019). Accumulated evidence demonstrated that social cognition has independent neural substrates and functions from general cognitive function (see Van Overwalle, 2009 for a review). Social cognitive dysfunction (SCD) is a core cognitive phenotype for diverse clinical conditions (for reviews, see Henry et al., 2016; Cotter et al., 2018). The literature of surgery related SCD highlights the separability of SCD and general cognitive deficits in patients. For example, emotion recognition deficits occurred in patients after glioblastoma surgery, while their object recognition did not change (Sinha et al., 2020). A review of patients who had undergone temporal lobe epilepsy surgery summarized that most studies that reported IQ scores did not show significant results regarding general cognition and social cognition (Mikula et al., 2021). However, previous studies always involve brain surgery, thus, it is unclear that whether their SCD was caused by anesthesia. Recently, one study provided new insights into SCD in patients after anesthesia and surgery, which assessed the occurrence rate of SCD after anesthesia and surgery and suggested that SCD after anesthesia and surgery was also independent of general cognitive deficits (Zhang et al., 2020).

Specifically, the recent study on SCD after anesthesia and surgery employed a biological motion (BM) perception task to evaluate the patients’ social cognitive ability (Zhang et al., 2020). BM is the movement of animate entities, such as walking, knocking, and throwing by humans. BM processing capability has been considered a hallmark of social cognition and the core of engaging in everyday social interactions (Blake and Shiffrar, 2007; Pavlova, 2012; Troje, 2012; Gao et al., 2016; Steel et al., 2016). Our brain has a sophisticated capability in processing BM, which can be conspicuously demonstrated *via* a limited set of point-lights (e.g., 13 points) placed at distinct joints of the human body (Johansson, 1973). Zhang et al. (2020) tested an essential characteristic of BM perception: the global processing of BM, reflected by the ability that recognizes the BM from scrambled masks. They found 31.25% of patients exhibited BM perception deficits after anesthesia and surgery. Moreover, there was no correlation between BM perception deficits and Mini-Mental State Examination scores, which evaluate the general cognitive deficits. This study revealed the existence of SCD after anesthesia and surgery, however, it only assessed one aspect of social cognition. As social cognition contains multiple facets (Henry et al., 2016), additional evidence is needed for the other social cognitive domain.

The current study tested another typical aspect of social cognition: emotion recognition, addressing the ability to discriminate between the emotional states of others through their expressions (Frith, 2008; Frith and Frith, 2012). The emotion recognition deficits have been extensively studied in patients with SCD (Kumfor and Piguet, 2013; Henry et al., 2016; Cotter et al., 2018), and such deficits could impair an individual’s mental health dramatically (Kee et al., 2003; Finset, 2012; Cacioppo et al., 2014; Hasson-Ohayon et al., 2017). Although emotions are mainly extracted from facial expressions, it is widely agreed that humans can recognize emotions from others’ BM (Okruszek, 2018). Probing emotion perception using dynamic BM stimuli well captures the complexities of day-to-day social interactions (Pavlova., 2012). Emotional point-light BM has been employed as a useful method of studying SCD in patients with neurodegenerative disease (e.g., schizophrenia, Alzheimer’s dementia, and Parkinson’s disease; see Okruszek, 2018 for a review). Consequently, in the present study, we determine to employ the emotional point-light BM as stimuli.

The present study investigated whether BM emotion perception was impaired after anesthesia and surgery. We employed an emotional BM recognition task that required the patients select the corresponding action label and emotion label for a perceived point-light BM with three possible types of action and emotions. This task could test the emotional perception of BM and meanwhile ensure normal BM recognition capacity. We compared the labeling performances of patients between pre-operation and post-operation. If only the emotion labeling performances were impaired after anesthesia and surgery, but the action labeling performances retained a consistent level as pre-operation, we would conclude that the patients had deficits in emotion perception of BM. Otherwise, if both the emotion labeling and the action labeling performances were impaired after anesthesia and surgery, the deficits in emotion labeling would possibly be driven by the impairment of BM recognition capacity and not be concluded as emotion recognition dysfunction.

## Materials and methods

### Study design and participants

A total of 227 patients who underwent cardiac surgery in the First Affiliated Hospital of College of Medicine, Zhejiang University (Hangzhou, China) were enrolled. Patients’ age was in the range of 40–72 years old, and their American Society of Anesthesiologists (ASA) status was less than or equal to class III. Of the 227 patients, 167 patients were excluded from the study due to incomplete data: 2 patients died before surgery; 3 patients decided to abandon the surgery; 19 patients attempted to abandon the examination preoperatively; 70 patients left the examination postoperatively; 60 patients were discharged before conducting the test postoperatively; 13 patients declined to undergo cardiopulmonary bypass (CPB). A total of 60 patients (33 male and 27 female) completed the study, and their average age was 54.9 ± 8.5 years old. The study protocol was approved by the Ethics Committee of Zhejiang University. The clinical trial registration number is ChiCTR2200058772. The date of registration is April 16, 2022. Written informed consent was obtained from all participants.

### Surgery and anesthesia

All patients underwent elective cardiac surgery with CPB. The clinical indicators, such as electrocardiogram, invasive arterial blood pressure, pulse oximetry, central venous pressure, capnography, temperature, and depth of anesthesia were collected. Anesthesia was induced with midazolam (0.04 mg/kg), etomidate (0.3 mg/kg), and sufentanil (1 μg/kg), and muscle relaxation was achieved with administration of cisatracurium (0.2 mg/kg). Anesthesia was conducted using propofol (50–100 μg/kg·min) and atracurium (0.1 mg/kg·h), and patients intermittently received midazolam (0.04 mg/kg) and sufentanil (0.5 μg/kg) through the vein to maintain the appropriate anesthetic depth. After the induction of anesthesia, an urinary catheter, nasopharyngeal temperature probes, and rectal temperature probes were placed.

After the administration of an initial bolus of heparin (300–400 IU/kg) to maintain the activated clotting time above 480 s, CPB was initiated and maintained according to a strict protocol with standardized cannulation sites, blood gas management, pump flow, and temperature targets. In addition, the mean arterial pressure was measured at 15–30 min after CPB, in order to maintain the partial pressure of arterial oxygen at 150–250 mmHg and the partial pressure of arterial carbon dioxide at 35–40 mmHg using the alpha-stat approach. The blood cardioplegia was set to a ratio of 1:4 for myocardial protection. Perfusion was maintained at pump flows of 2.2–2.4 l/min/m^2^ to maintain the MAP of 50–80 mmHg. The oxygenators were obtained from Sorin Biomedica (Mirandola, Italy). The pumps were all of SIII grade (Stockert, Munich, Germany). Red blood cells were transfused to maintain a hematocrit of 20–25% on CPB. Once systemic temperature (central) reached 36°C, weaning from CPB was conducted using a previously described protocol. Systolic blood pressure was maintained at 100–130 mmHg. Arterial blood gasses were measured at 30-min intervals to maintain the partial pressure of arterial oxygen at 150 mmHg and the partial pressure of arterial carbon dioxide at 35–40 mmHg. Red blood cells were transfused to maintain a hematocrit of 25–30%. Protamine was accordingly applied after weaning from CPB.

### BM recognition task

**Figure 1:**
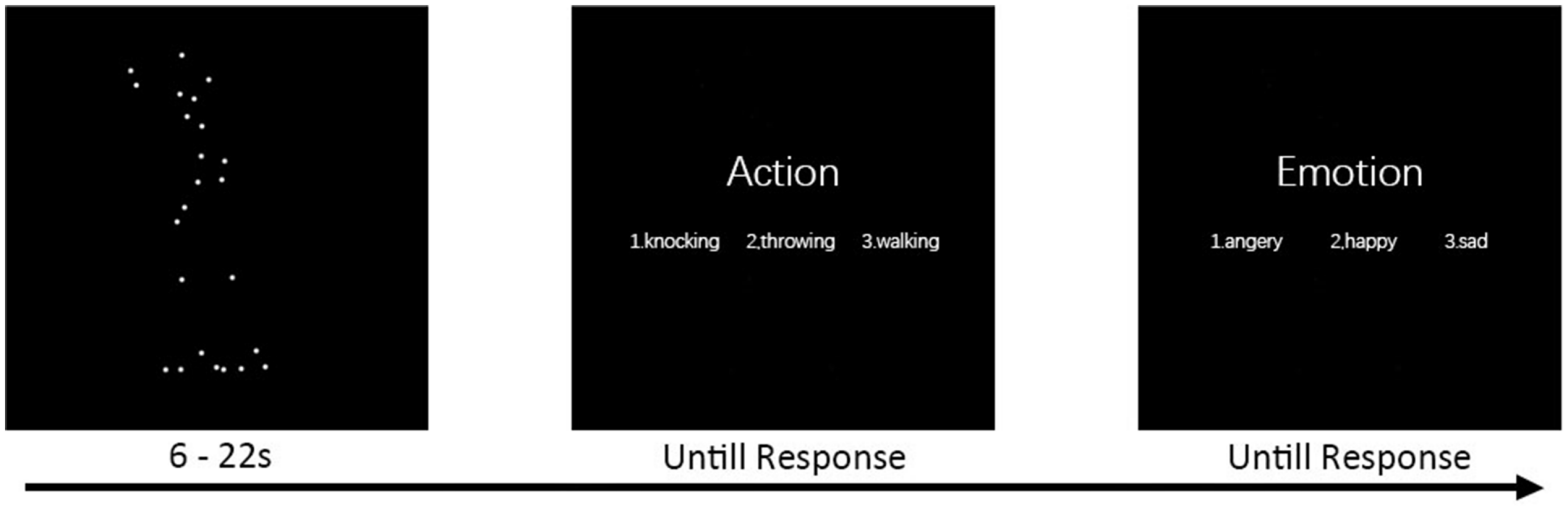
BM recognition task. Each trial began with the presentation of a point-light BM stimulus. The stimulus lasted for 6–22 s, depending on the duration of action. Patients were asked to report the labels of action and emotion of the BM. Two questions followed the BM sequentially, and an experimenter entered the selected label for the patient.

The BM recognition task was run on a Lenovo Yoga 900 laptop. The screen size of this laptop was 13.3 inches, with a resolution of 3,200 × 1800 and a refresh rate of 60 Hz. Patients were placed 60 cm away from the screen of the laptop. The task lasted for approximately 30 min and consisted of 18 trials with the same procedure but different BM stimuli (Figure 1). In each trial, a point-light BM was presented at the screen center within a region with the visual angle of approximately 20° × 10°. All dots had the same size (0.28° × 0.28°) and color (white, RGB, [255,255,255]). The background color was black (RGB, [0,0,0]). The BM stimulus lasted 6–22 s, depending on the duration of action. Patients were asked to orally report the labels of actions (from walking, knocking, or throwing) and emotions (from happiness, anger, or sadness) of the BM. The BM stimuli in each trial were evenly distributed among the three types of actions and the three types of emotions. After the BM presentation, the words “action” and “emotion” (in Chinese) were presented sequentially at the screen center, with the three possible labels listed below. An experimenter entered the selected label for each patient. Patients who did not report the labels during the BM presentation were encouraged to select the labels at this stage. Patients received 6 practical trials with no feedback to familiarize themselves with the procedure before the formal experiment.

### Data collection and analysis

The BM recognition task was conducted 1 day before the surgery (pre-operative condition) and 7 days after the surgery (post-operative condition). Patients were assigned to the SCD group and the non-SCD group by the 1 standard deviation (SD) criterion, that is, the decrease of 1 SD from the baseline mean (Evered et al., 2011; Zhang et al., 2020). Such 1 SD criterion is mainly used to measure delayed neurocognitive recovery without a control group (Evered et al., 2011), and it was utilized by Zhang et al. (2020) to measure SCD. According to the 1 SD criterion, we calculated the differences in accuracy of emotion labeling between pre-operative and post-operative conditions. If the reduction of a patient’s emotion labeling accuracy was higher than 1 SD of pre-operative accuracy of all patients, that patient was diagnosed with SCD.

We performed a mixed-design analysis of variance (ANOVA) on the accuracy of action labeling and emotion labeling separately by taking time (pre-operative vs. post-operative) as the within-subject factor and group (SCD vs. non-SCD) as the between-subject factor. In the statistical analysis, except as otherwise noted, the data were normally distributed and had homogeneous variance. The alpha level of all tests was set to 0.05.

Pre-operative demographic and clinical data were collected, including age, educational level, gender, diabetes, hypertension, history of smoking, history of alcohol consumption, operation time, CPB duration, time of intensive care unit discharge, and time of hospital discharge. The demographic and clinical data were compared between SCD and non-SCD groups using the independent-samples *t*-test and Fisher’s exact test.

## Results

According to the 1 SD criterion, 11 (5 male and 6 female) of 60 patients (18.3%) met the criterion of SCD and were assigned to the SCD group, and the other 49 (28 male and 21 female) patients were assigned to the non-SCD group.

### Emotion labeling performance

**Figure 2:**
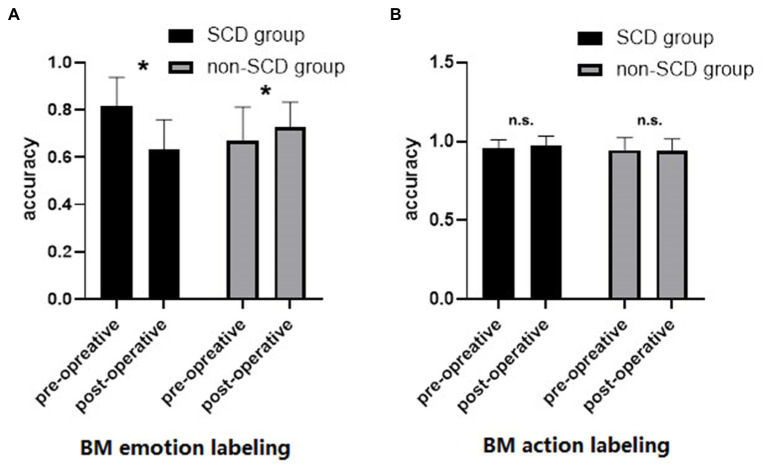
Comparison of the accuracy of BM emotion labeling **(A)** and BM action labeling **(B)**.

Figure 2A shows the accuracy of BM emotion labeling under each group and time points. The ANOVA revealed a significant main effect of time [*F*(1,58) = 12.198, *p* < 0.001, η^2^ = 0.034], suggesting that the overall accuracy of BM emotion labeling decreased after surgery. The main effect of group was not significant [*F*(1,58) = 0.554, *p* = 0.460, η^2^ = 0.006], indicating that the accuracies of BM emotion labeling were comparable between the two groups. Critically, there was a significant interaction between time and group [*F*(1,58) = 43.858, *p* < 0.001, η^2^ = 0.123]. Simple-effects analysis revealed that the accuracy of BM emotion labeling in the SCD group was significantly higher in pre-operative condition (accuracy = 0.818) than post-operative condition (accuracy = 0.631) [*t*(10) = 22.112, *p* < 0.001]. On the contrary, the accuracy of BM emotion labeling in the non-SCD group was significantly lower in pre-operative condition (accuracy = 0.668) than in post-operative condition (accuracy = 0.726) [*t*(58) = −3.344, *p* = 0.002], indicating a practice effect.

### Action labeling performance

Figure 2B shows the accuracy of BM action labeling in each group and time. The ANOVA revealed no significant main effect of time [*F*(1,58) = 0.406, *p* = 0.527, η^2^ = 0.002] or main effect of group [*F*(1,58) = 1.021, *p* = 0.316, η^2^ = 0.012]. The interaction between time and group was not statistically significant [*F*(1,58) = 0.801, *p* = 0.374, η^2^ = 0.004]. These results suggested that BM action recognition was not affected by anesthesia and surgery.

#### Comparison of demographic and clinical data between SCD and non-SCD groups

The demographic and clinical data were compared between SCD and non-SCD groups (Table 1). There was no significant difference in the demographic and clinical data between the SCD and non-SCD groups except for the CPB duration. The CPB duration in the SCD group was significantly longer than in the non-SCD group. The accuracy of BM emotion labeling was decreased when CPB duration was prolonged. These results demonstrated that emotion recognition dysfunction was related to anesthesia.

**Table 1:** Comparison of the demographic and clinical data (mean ± SD) of patients.

	All patients (*n* = 60)	SCD (*n* = 11)	Non-SCD (*n* = 49)	Comparison between SCD and non-SCD
Age (years old)	54.9 ± 8.5	56.1 ± 8.9	54.6 ± 8.5	*t*(58) = −0.512, *p* = 0.611
Educational level (years)	9.0 ± 3.4	8.2 ± 2.4	9,1 ± 3.5	*t*(58) = 0.857, *p* = 0.395
Gender (male/female)	33/27	5/6	28/21	*p* = 0.205
Diabetes	30%	36.4%	28.6%	*p* = 0.241
Hypertension	1.7%	9.1%	0%	*p* = 0.183
History of smoking	30%	45.5%	26.5%	*p* = 0.131
History of alcohol consumption	26.7%	27.3%	26.5%	*p* = 0.289
Surgery duration (min)	217.7 ± 74.9	249.5 ± 78.6	210.5 ± 73.0	*t*(58) = −1.577, *p* = 0.120
CPB duration (min)	107.6 ± 42.0	132.9 ± 37.4	101.9 ± 41.2	*t*(58) = −2.291, *p* = 0.026
Intensive care unit duration (day)	3.4 ± 0.7	3.4 ± 0.7	3.4 ± 0.7	*t*(58) = −0.228, *p* = 0.775
Time of hospital discharge (day)	14.3 ± 4.2	13.5 ± 3.0	14.5 ± 4.4	*t*(58) = 0.668, *p* = 0.507

## Discussion

In the present study, we investigated whether emotion recognition was impaired after anesthesia and surgery. We found that 18.3% of patients exhibited impaired emotion perception compared with before cardiac surgery, while their action perception was intact. These results suggested that SCD after anesthesia and surgery was not limited to a specific aspect of social cognition.

The present study contributed to fully understanding the impact of surgery on emotion recognition. Our finding was congruent with previous studies that impairments in emotion recognition could be present in patients after surgery (e.g., Amlerova et al., 2014; Campanella et al., 2015; Sinha et al., 2020; Buunk et al., 2022). However, previous studies that investigated surgery-related emotion recognition deficits always involve brain surgery (e.g., glioblastoma surgery, temporal lobe epilepsy surgery). The present study was the first to demonstrate the influence of anesthesia and surgery on emotion recognition in patients without brain surgery, highlighting the effect of anesthesia during surgery. Another difference between the present and previous studies in patients after surgery was the stimulus types. Previous studies mainly used emotional faces as emotion recognition task stimuli (Mikula et al., 2021). The present study was the first to explore BM emotion recognition in patients after surgery. Body movements were also a source of emotionally relevant information (Nackaerts et al., 2012), while hardly used in the studies of patients after surgery. Considering the dynamic nature of social cognitive processes, point-light BMs have relatively high ecological validity in evaluating patients’ social cognition ability (Okruszek, 2018). The present study provides an essential complement to understanding emotional recognition disorders in patients after surgery.

Together with Zhang et al. (2020), the present study offered empirical evidence that social cognition was impaired in a group of patients after anesthesia and surgery. The incidence of SCD after anesthesia and surgery was 18.3% in the present study. It was lower than Zhang et al. (2020) that 31.25% of patients exhibited SCD after anesthesia and surgery. The source of different incidences may come from two aspects. First, these studies employed different tasks. The present study employed a BM emotion recognition task that required the participants to select the emotion label for the perceived point-light BM. The task measures an essential social cognition ability that distinguishes basic emotional categories. Zhang et al. (2020) employed a BM detection task that required the participants to discriminate whether there is a point-light BM in dynamic noise. This task might be more fragile. Second, the patients’ ages were older in the previous (66.8 ± 4.8 years old) than in the present (54.9 ± 8.5 years old) study, suggesting that older patients are at greater risk for SCD after anesthesia and surgery. In addition, the present study did not reveal a deficit in BM action perception. We thought that this finding was not inconsistent with Zhang et al. (2020). In their study, the deficit in BM perception was reflected by an inversion effect, which referred to the impairment of BM perception by inverting the BM stimuli. The present study tapped a different aspect of BM perception: evaluating the types of BM actions. However, a limitation should be noted: the accuracy of BM action perception was relatively high and could result in the lack of sensitivity to detect change.

We designed the BM recognition task in order to test emotional perception of BM and meanwhile ensure the existence of normal BM recognition capacity. In this task, the accuracy of emotion perception and the accuracy of action perception came from the same BM perception process rather than two different tasks with potentially unmatched levels of difficulties. Thus, the results could reflect that the emotion recognition had specific dysfunction and was not driven by the impairment of BM recognition capacity. The emotion perception accuracy of the non-SCD group was unexpectedly increased after surgery. This result suggested that the normal population could improve their emotion perception accuracy by practice, which conversely supported that the impairment of emotion perception in the SCD group was indeed caused by anesthesia and surgery.

Someone may argue that the occurrence of SCD was influenced by not only anesthesia and surgery but some confounding variables. For example, anxiety, depression, stress, or personality type were mismatched between the SCD group and the non-SCD group. We argue that this alternative would not explain our findings. Participants were assigned to the SCD group and the non-SCD group by performance differences between pre-operative and post-operative conditions. If the mismatches exist between the SCD group and the non-SCD group, they would exist both in pre-operative and post-operative conditions and not cause a difference. Thus, we were not likely to overestimate the incidence of emotion recognition dysfunction after anesthesia and surgery.

Social perceptual failures are mainly manifested as difficulties in the identification of others’ emotions (Henry et al., 2016). Therefore, revealing a deficit in patients’ emotional perception after anesthesia and surgery has an important clinical significance. Our study provided a new approach for assessing social cognition in patients undergoing surgery, which is easy to perform, and user-friendly for patients. However, the present study only concentrated on cardiac surgery. It has remained elusive whether the findings of this study could be extended to other types of surgery. Further studies could also investigate how individual characteristics influence postoperative emotion recognition dysfunction. For example, whether empathy and autism-spectrum quotient, which are the factors often considered in social cognition studies (e.g., Gao et al., 2016; Liu et al., 2018), can predict the occurrence of postoperative emotion recognition dysfunction. Additionally, we found a significant difference in CPB duration between the SCD and the non-SCD groups, indicating that the CPB may play a moderating role in SCD. This result requires to be verified in future studies.

## Data availability statement

The original contributions presented in the study are included in the article/Supplementary material, further inquiries can be directed to the corresponding authors.

## Ethics statement

The studies involving human participants were reviewed and approved by Clinical Research Ethics Committee of the First Affiliated Hospital, College of Medicine, Zhejiang University. The patients/participants provided their written informed consent to participate in this study.

## Author contributions

DZ, YS, ZC, YG, JH, and ZG conceived and designed the study. DZ, YS, ZC, YG, JH, and XL performed the experiments. DZ, YS, ZG, JH, and XL wrote the manuscript. All authors analyzed the data, reviewed the manuscript, and approved the final version of the manuscript for submission.

## Funding

This research was supported by Basic Public Welfare Project of Zhejiang Province, China (Grant No. LGF19H090023), Medical and Health Science and Technology Project of Zhejiang Province, China (Grant No. 2018KY064), and Fundamental Research Funds for the Central Universities.

## Conflict of interest

The authors declare that the research was conducted in the absence of any commercial or financial relationships that could be construed as a potential conflict of interest.

## Publisher’s note

All claims expressed in this article are solely those of the authors and do not necessarily represent those of their affiliated organizations, or those of the publisher, the editors and the reviewers. Any product that may be evaluated in this article, or claim that may be made by its manufacturer, is not guaranteed or endorsed by the publisher.
